# Single-shot BOTDA based on an optical chirp chain probe wave for distributed ultrafast measurement

**DOI:** 10.1038/s41377-018-0030-0

**Published:** 2018-07-11

**Authors:** Dengwang Zhou, Yongkang Dong, Benzhang Wang, Chao Pang, Dexin Ba, Hongying Zhang, Zhiwei Lu, Hui Li, Xiaoyi Bao

**Affiliations:** 1 National Key Laboratory of Science and Technology on Tunable Laser, Harbin Institute of Technology, 150001 Harbin, China; 2 Department of Optoelectronic Information, Science and Engineering, Harbin University of Science and Technology, 150080 Harbin, China; 3 School of Civil Engineering, Harbin Institute of Technology, 150001 Harbin, China; 4 Fiber Optics Group, Department of Physics, University of Ottawa, Ottawa, ON K1N 6N5 Canada

## Abstract

Brillouin optical time-domain analysis (BOTDA) requires frequency mapping of the Brillouin spectrum to obtain environmental information (e.g., temperature or strain) over the length of the sensing fiber, with the finite frequency-sweeping time-limiting applications to only static or slowly varying strain or temperature environments. To solve this problem, we propose the use of an optical chirp chain probe wave to remove the requirement of frequency sweeping for the Brillouin spectrum, which enables distributed ultrafast strain measurement with a single pump pulse. The optical chirp chain is generated using a frequency-agile technique via a fast-frequency-changing microwave, which covers a larger frequency range around the Stokes frequency relative to the pump wave, so that a distributed Brillouin gain spectrum along the fiber is realized. Dynamic strain measurements for periodic mechanical vibration, mechanical shock, and a switch event are demonstrated at sampling rates of 25 kHz, 2.5 MHz and 6.25 MHz, respectively. To the best of our knowledge, this is the first demonstration of distributed Brillouin strain sensing with a wide-dynamic range at a sampling rate of up to the MHz level.

## Sensors: Lighting the way to low-cost, ultrafast sensors

Scientists have developed an inexpensive and ultrafast sensor that could be used in a range of applications from monitoring geo-engineering to the structural integrity of large infrastructure. Compared with conventional electrical sensing, optical fiber sensors are much smaller and cheaper, immune to external electromagnetic interference and can offer millions of sensing points simultaneously. However, processing the information provided by optical fibers can be time-consuming, limiting their applications. Now, a team of researchers from China and Canada, led by Yongkang Dong from Harbin Institute of Technology in China, has developed an innovative technique that generates a probe wave comprising short optical chirps that can be quickly demodulated by injecting a single-shot pump pulse into the fiber. The work paves the way for ultrafast sensors for use in applications from the infrastructure to geophysical research.

## Introduction

In modern industry^1–3^, geophysical research^4–6^, the health monitoring of civil infrastructures^1,2^, and the motion capturing of robot hands^7,8^ and the human body^9^, a truly distributed ultrafast measurement, are widely required for real-time monitoring of distributed strain or temperature information. Compared with conventional electric sensing networks, optical fiber sensing offers an attractive solution due to several advantages: smallness in size, low cost, long sensing range, chemical inertness, and immunity to external-electromagnetic interference^10^, where the optical fiber simultaneously acts as the optical transmitting medium and millions of sensing points.

Brillouin-based distributed sensors have been widely reported since the 1990s^2,11,12^. Generally, two counter-propagating optical waves (i.e., pump wave and probe wave) are launched into a fiber under test (FUT). As the frequency difference between these two waves approaches the local Brillouin frequency shift (BFS) of the FUT, the optical power transferred from the high-frequency light wave to the low-frequency light wave reaches a maximum. As a result, the Brillouin gain spectrum (BGS) can be obtained by sweeping the detuned frequency. Then, the BFS, which is dependent on both applied strain and temperature along the FUT^2,10,11,13–15^, can be calculated by curve fitting the BGS. The process of frequency tuning to obtain the BGS is time-consuming. Distributed fast strain measurement has been previously realized using several techniques: Brillouin optical correlation-domain analysis (BOCDA)^3,16–18^, Brillouin optical correlation-domain reflectometry (BOCDR)^3,19–25^, and Brillouin optical time-domain analysis (BOTDA)^12,26–32^.

By employing a BOCDA with optimized time gates and an unbalanced Mach–Zehnder delay line^33^, a vibration measurement with a frequency of 200 Hz and 10-cm spatial resolution in a 20-m measurement range is realized at a 1-kHz sampling rate. The sampling rate can be further improved to 5 kHz based on random accessibility and the use of a lock-in amplifier^34^. An improved BOCDR scheme^20^ is implemented by utilizing a high-speed voltage-controlled oscillator to convert the BFS to a phase delay, resulting in a 100-kHz sampling rate at a single point and 100-Hz repetition rate at 1000 points with 10 times averaging. In the BOCDA/BOCDR techniques, the high-speed sampling rate for dynamic strain at a single point is obtained at the expense of distribution due to the slow sweeping peak location process, which requires priori information for the strain location.

Remarkably, BOTDA has been widely studied because of its superior performance for long-distance-distributed measurement^27,35–38^, wide-strain dynamic range^12,31^, and high-speed sampling rate^12,30,31,39–41^. In a conventional BOTDA system, the distributed BGS is reconstructed by injecting a high-power pump pulse to interrogate the local-stimulated Brillouin scattering (SBS) and then sweeping the continuous probe wave frequency over a wide spectral range; such a process imposes the main limiting factor on the sampling rate^42^. To date, BOTDA for dynamic measurement is classified into three categories: frequency-comb-based sweep-free scheme^43–47^, slope-assisted scheme^30,31,39,42,48,49^, and fast-frequency sweeping scheme^50,51^. The sweep-free scheme makes use of a frequency comb to demodulate the BGS; however, there is a trade-off between the spatial resolution and the frequency interval for the comb, with the spatial resolution usually a few tens of meters. In the slope-assisted scheme, the frequency detuning between pump and probe waves is set at the middle of the slopes for the BGS^39,40,42^, Brillouin phase-shift spectrum^30,52^ or their ratio spectrum^31,49^, so that a single pump pulse can demodulate the distributed BFS along the fiber. It is noted that the sampling rate is only limited by the length of the FUT without averaging, but the dynamic range is restricted by the linewidth of the BGS. Several improved schemes have been introduced to improve the dynamic range to 5000 με by use of the frequency-agile technique^12,31,53^ at the expense of the sampling rate. The last scheme is realized by compressing the frequency switching time for the probe wave. A 100-Hz vibration measurement in a 100-m FUT based on a fast BOTDA^50^ is acquired by the frequency-agile technique to quickly switch 100 scanning frequencies for the probe wave corresponding to 100 synchronous pump pulses, resulting in a sampling rate of ~10 kHz, with the distributed BGS tailored from the time trace. Obviously, the sampling rate for this scheme depends on both the length of the FUT and the number of scanning frequencies. Recently, a frequency-sweep pulsed BOTDA^51^ was implemented to obtain a Lorentzian-shaped correlated gain in the time domain by linear-frequency modulation of two 1.0-μs pulses as pump and probe pulses coupled with a point (20 m) dynamic strain measurement with a sampling rate of 10 kHz obtained at the end of 10 km. Although a distributed dynamic measurement can be realized through adjusting the delay between the probe and pump pulses, the effective sampling rate is divided by the sensing point number.

In this paper, we theoretically and experimentally report a novel single-shot BOTDA for distributed ultrafast measurement based on optical chirp chain (OCC), named OCC–BOTDA, where the probe wave is frequency modulated into short optical chirp segments and then cascaded into an OCC. Only a single-shot pump pulse is required to recover the distributed BGS along the OCC probe wave when the local BFS is covered by the wide-frequency span between the pump pulse and the OCC probe wave. The maximum sampling rate can reach the order of MHz, which is only restricted by the length of the FUT. The dynamic strain range can be readily tuned by setting the frequency span. We further test the distributed ultrafast performance of this system: a periodic mechanical vibration is monitored with a 25-kHz sampling rate; a mechanical shock measurement is designed by rapidly stretching a 2-m fiber section via knocking a rotating mechanism, which is captured at a sampling rate of 2.5 MHz; a switch event is designed to simulate a fast 20-MHz BFS change, which is recorded at a sampling rate of 6.25 MHz.

## Materials and methods

### Operation principle

A typical BOTDA system is based on a “pump-probe” scheme^2,28,31,54–56^ such that the power transfer from the optical pump pulse to the continuous probe wave occurs through SBS when their frequency difference is set near the BFS of the FUT. The evolution of these two waves (indicated by *A*_p_ and *A*_s_, respectively) and an acoustic wave (indicated by *Q*) is described by the following coupled-wave equations^2,57^

1 $$\begin{array}{l}\frac{{\partial A_{\rm{p}}}}{{\partial z}} + \frac{n}{c}\frac{{\partial A_{\rm{p}}}}{{\partial t}} = \frac{{i\omega \gamma _{\rm{e}}}}{{2nc\rho _0}}QA_{\rm{s}}\\ \frac{{\partial A_{\rm{s}}}}{{\partial z}} - \frac{n}{c}\frac{{\partial A_{\rm{s}}}}{{\partial t}} = - \frac{{i\omega \gamma _{\rm{e}}}}{{2nc\rho _0}}Q ^\ast A_{\rm{p}}\\ \left( {\Gamma _{\rm{B}} - 2i\Omega } \right)\frac{{\partial Q}}{{\partial t}} + \left( {\Omega _{\rm{B}}^2 - \Omega ^2 - i\Omega \Gamma _{\rm{B}}} \right)Q = \varepsilon _0\gamma _{\rm{e}}q^2A_{\rm{p}}A_{\rm{s}}^ \ast \end{array}$$

where *ω* ≡ 2*π*⋅*ν*_p_ ≈ 2*π*⋅*ν*_s_ is the angular frequency of the optical waves, *n* is the refractive index of the FUT core, *c* is the speed of light in the vacuum, *γ*_e_ is the electrostrictive coefficient, *ρ*_0_ denotes the mean density of the FUT core, *ε*_0_ is the vacuum permittivity, and Γ_B_ = 1/*τ*_p_ is the Brillouin gain linewidth. Ω = 2*π*(*ν*_p_ − *ν*_s_), *q* = *k*_p_ + *k*_s_. Ω_B_ = 2*π*⋅*f*_BFS_, where *f*_BFS_ = 2*nv*_p_*V*_a_/*c* is the local BFS of the FUT and *V*_a_ is the speed of the moving acoustic wave. Generally, the value of the BFS is ~11 GHz for a single-mode fiber at the communication wavelength of 1550 nm^2,57^.

**Fig. 1:**
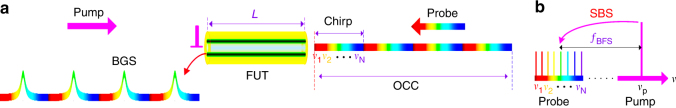
The operation principle of an OCC-BTODA. **a** The OCC probe wave is temporally cascaded by several short optical chirp segments in which the frequency is scanned from *v*_1_ to *v*_N_, while the pump pulse requires just single shot to obtain the distributed BGS along the FUT. **b** The frequency relationship between the pump pulse and the continuous probe wave

The operation principle for the OCC–BOTDA is illustrated in Fig. 1a. The optical probe wave is frequency modulated from*ν*_1_ to *ν*_N_(N is the frequency number) in a few tens of nanoseconds, resulting in a short optical chirp segment, which can be regarded as the temporal compression of the traditional sweeping frequency method. Then, the probe wave is composed of several short optical chirp segments, which are referred to as the optical chirp chain (OCC). It is noted that the OCC has two types of on-link modes: one is the sawtooth mode (two adjacent optical short-chirp segments are cascaded by a head-to-tail cohesion), and the other is the triangular mode (two adjacent optical short-chirp segments are cascaded by a head-to-head or tail-to-tail cohesion). Similar to typical BOTDA systems, the pump wave is shaped into a pulse pattern, with a width that is narrower than the duration of the short optical chirp segment. The OCC probe wave and the pump pulse are injected into an FUT in the opposite direction. When the frequency difference between the pump pulse and the OCC probe wave matches the BFS for the FUT (Fig. 1b), the BGS can be recovered along the short optical chirp segment in the time domain due to the Brillouin amplification.

**Fig. 2:**
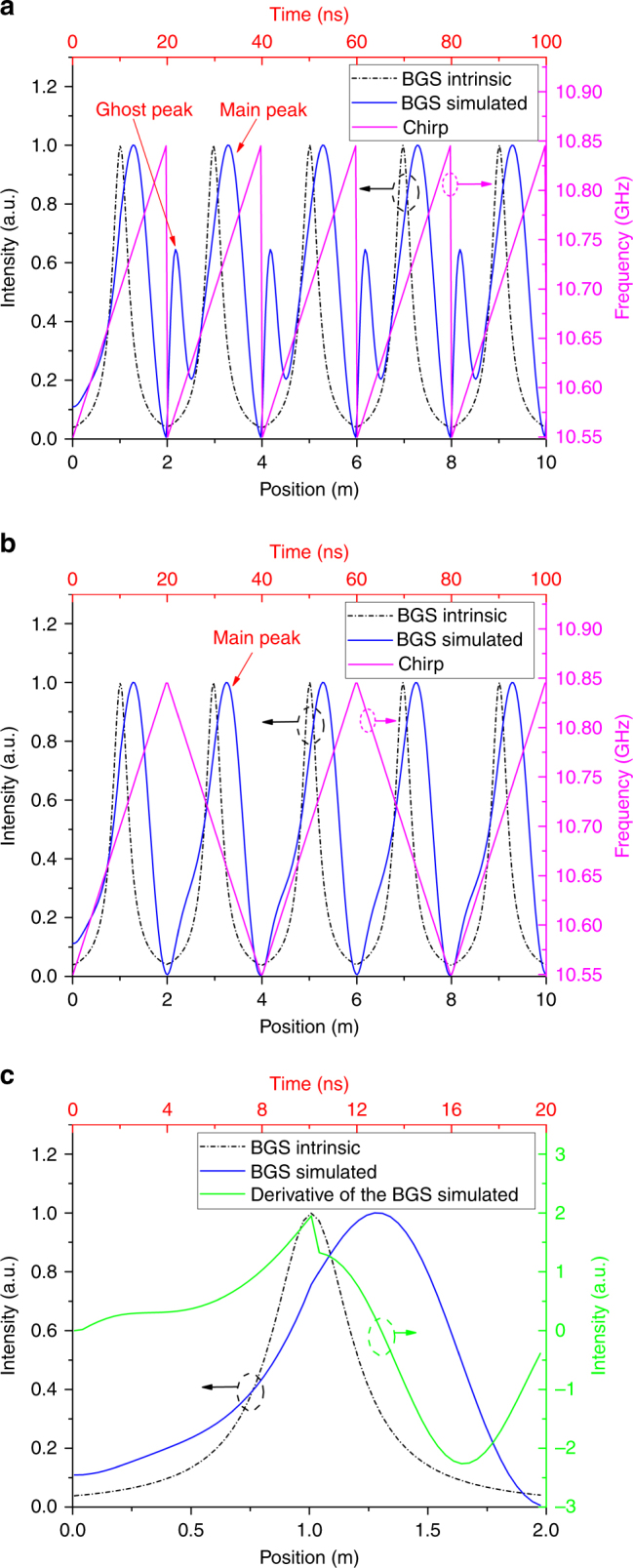
Simulation of the OCC–BOTDA. **a** The distributed BGSs along the fiber for the intrinsic case (black dashed-dotted line), “sawtooth” mode (blue line), and distributed chirp frequency (magenta line). **b** The distributed BGSs along the fiber for the intrinsic case (black dashed-dotted line), “triangular” mode (blue line), and distributed chirp frequency (magenta line). **c** The intrinsic BGS (black dashed-dotted line), simulated BGS (blue line), and derivative of the simulated BGS (green line) within the first 2 m

The OCC–BOTDA system is numerically calculated based on Eq. (1), with the simulation results corresponding to the two chirp modes (magenta line), i.e., “sawtooth” mode and “triangular” mode shown in Fig. 2a, b, respectively. Here, the duration of the short optical chirp segment is set at 20 ns, with 300 MHz of frequency coverage, while the width of the pump pulse is set at 10 ns. A 10-m-long fiber with *f*_BFS_ = 10.70 GHz and Γ_B_/2*π* = 30 MHz is used. It is clearly illustrated that the intrinsic BGSs (black dashed-dotted line) along the fiber describes a symmetrical Lorentzian profile as the frequency of the OCC is scanned from 10.550 GHz to 10.845 GHz in every 2-m segment. Compared with the intrinsic BGS, in both the “sawtooth” mode and “triangular” mode, the simulated BGS exhibits an asymmetrical and broadened profile, while its peak shows a time delay of ~2.7 ns (or position shift) corresponding to a frequency shift of ~41 MHz. Such a phenomenon arises from the transient SBS interaction, i.e., the hysteresis effect due to Brillouin amplification with respect to the acoustic wave excitation. As shown in Fig. 2c, the peaks of the intrinsic BGS and the derivative of the simulated BGS (green line) show complete coincidence, which indicates that the growth rate of the acoustic wave reaches a maximum for the resonant frequency and then decreases due to the increased detuning from the BFS; subsequently, the measured BGS exhibits a time delay with respect to the intrinsic case.

Note that a “ghost peak” appears at the frequency discontinuity position in the “sawtooth” mode in Fig. 2a due to the generation of an equivalent frequency near the BFS when the frequency is decreased from the highest frequency component *ν*_N_ to the lowest frequency component *ν*_1_; the “ghost peak” disappears in the “triangular” mode in Fig. 2b due to the smooth convergence for the frequency in the link point between two short optical chirp segments. In the following experiment, the “sawtooth” mode OCC probe wave is adopted, since the demodulation process for the local BFS in the “sawtooth” mode is simpler than in the “triangular” mode.

### Sampling rate and spatial resolution

In contrast to the time-consuming process of frequency sweeping for traditional BOTDA, the distributed BGS along the FUT based on the OCC–BOTDA can be rapidly demodulated by only injecting a single-shot pump pulse into the fiber. Therefore, the maximum sampling rate of this scheme is given by

2 $$f_{\rm{Sa}} = \frac{1}{{N_{\rm{ave}} \cdot T}}$$

where *N*_ave_ is the number of averages, *T* = 2*nL*/*c* is the round-trip time for the sensing fiber with a length of *L*. It can be seen that without averaging, the maximum sampling rate is only limited by the FUT length.

The spatial resolution for this scheme is restricted by the duration of the short optical chirp segment ∆*T* and is given by

3 $$\Delta z_{\rm{SR}} = \frac{{c\Delta T}}{{2n}}$$

### Experimental setup

**Fig. 3:**
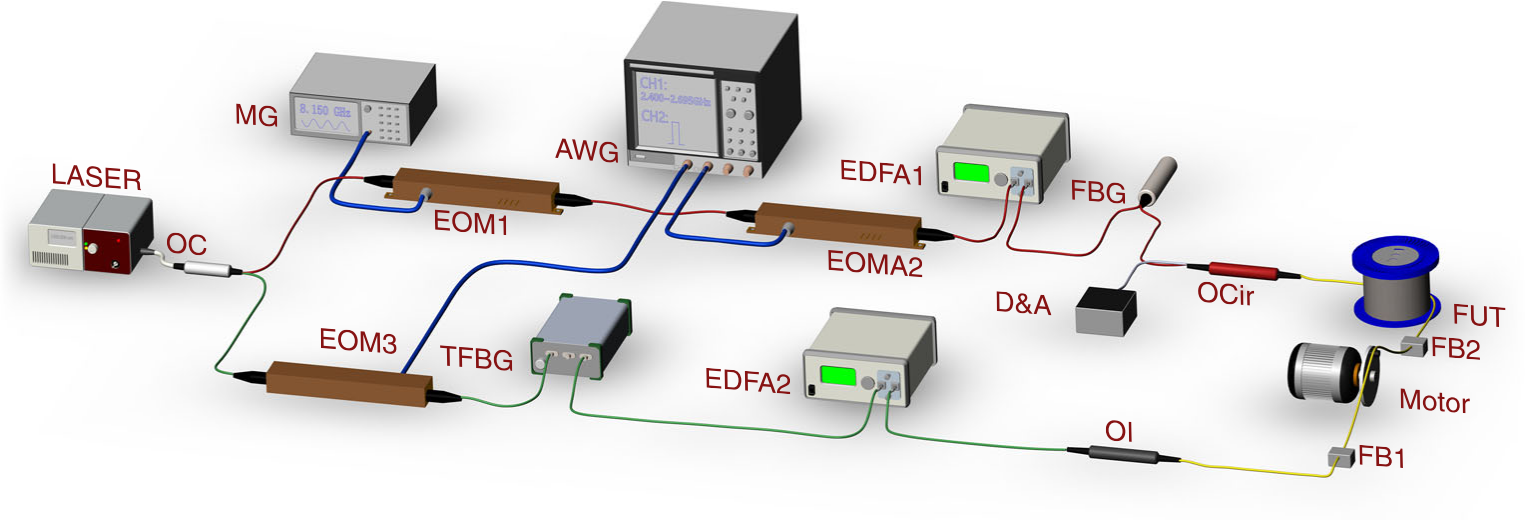
Experimental configuration for the OCC-BTODA system. MG microwave generator, EOM electro-optic modulator, AWG arbitrary-waveform generator, EDFA erbium-doped fiber amplifier, FUT fiber under test, TFBG tunable fiber Bragg grating, OC optical coupler, OI optical isolator, OCir optical circulator, D&A photodetector and data acquisition modules, FB fixed base. Note that the polarization for all the optical devices is aligned along the slow axis to eliminate polarization noise and fading

Figure 3 shows the experimental setup used to test the ultrafast performance of the OCC–BOTDA scheme. The frequency-agile technique involves generation of fast-frequency-changing microwaves by an AWG with a high bandwidth and high digital sampling rate, in which the output can be reconfigured by editing the AWG control program. The frequencies should satisfy the equation^53^

4 $$f_{\rm{AWG}}\left( t \right) = \, f_1 + \eta \cdot t$$

where *f*_1_ is the initial frequency. *η* = Δ*f*/Δ*T* is the frequency-changing rate, Δ*f* is the frequency span, and Δ*T* is the duration of the short optical chirp segment. Due to the bandwidth limitation for AWG in our laboratory, we chose the dual modulation^53^ to implement a wide-frequency span between the OCC probe wave and the pump pulse, which can cover the BFS of the FUT.

An optical fiber laser with a narrow linewidth of ~10 kHz was used as the light source, in which the output parameters were set at 1550 nm and 100 mW. Subsequently, the 90% branch (red line) for the optical coupler was selected to be chopped for the optical pulse as the pump wave. The light was frequency modulated using an electro-optic modulator1 in the carrier-suppressed regime, which is driven by an 8.15-GHz sinusoidal microwave output from a microwave generator. Then, the frequency-modulated light is intensity modulated using EOM2 driven by the CH2 of the AWG into a 10-ns optical pulse. After amplification to 1 W by an erbium-doped fiber amplifier1, an optical pulse with a higher-frequency sideband was selected through an optical fiber Bragg grating (FBG) filter. Then, the optical pulse was injected into the FUT through an optical circulator.

The lower branch with 10% component was used to generate an OCC for use as a probe wave. The light was frequency downshifted using EOM3 driven by the CH1 of AWG and a tunable FBG. Note that the output of CH1 was temporally cascaded by the sinusoidal microwave signals with a frequency span from 2.4 to 2.695 GHz and a frequency step of 5 MHz. As a result, a short optical chirp segment was generated with a width of 20 ns and frequency-changing rate of *η* = 15 MHz/ns. By using the “sawtooth” mode, the OCC probe wave was assembled and then amplified by EDFA2 to ~80 μW. Hence, both the pump pulse and the continuous probe wave were launched into the FUT. Finally, the Brillouin signal (i.e., amplified OCC-probe wave) was received by a D&A module at a sampling rate of 5 GS/s. To eliminate polarization noise and fading, the polarization of all optical devices was aligned along the slow axis, with a polarization maintaining fiber (PMF) with a BFS of 10.705 GHz used as the FUT. A photodetector with a bandwidth of 350 MHz and a response time of 2 ns in the D&A module was used to detect the OCC-probe wave.

## Results and discussions

### Static BGS measurement and twice-correlation algorithm

**Fig. 4:**
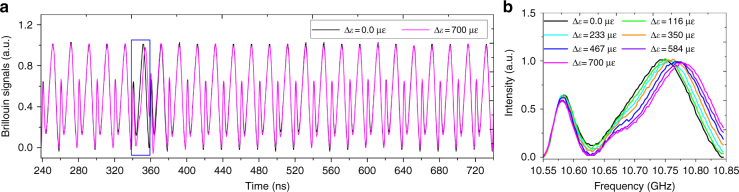
The static distributed BGS measurement based on the proposed OCC-BOTDA. **a** The time traces for the Brillouin signals for a strain change of ∆*ε* = 0.0 με and ∆*ε* = 700 με. **b** The zoom-in view for the BGSs for a strain change from ∆*ε* = 0.0 με to ∆*ε* = 700 με in the blue box. Averaging is carried out 200 times

The measured time traces of the Brillouin signal of the OCC–BOTDA system are shown in Fig. 4a. The data clearly show a BGS with a main peak and a ghost peak that is found to occur every 20 ns (corresponding to a spatial resolution of 2 m), which agrees well with the simulation results, as plotted in Fig. 2. Compared with the BGS (black line) for a strain change of ∆*ε* = 0.0 με, the BGS (magenta line within the blue wireframe) for a strain change ∆*ε* = 700 με is time-shifted. Subsequently, a zoom-in view for the blue wireframe is shown in Fig. 4b as the strain changes from ∆*ε* = 0.0 με to ∆*ε* = 700 με, with the horizontal axis label converted from a time value to a frequency value. It is clearly shown that the central frequency of the main peak initially shifts by ~42 MHz with respect to the local BFS at ~10.705 GHz at room temperature. The time (or frequency) of the main peak is evidently up-shifted as the strain change is increased.

However, it is very difficult to precisely calculate the BFS by typical Lorentzian or Gaussian fitting the main peak of the BGS due to its asymmetrical profile. Here, we propose a twice-correlation algorithm not only to make the measured BGS more symmetrical, but also to improve the signal-to-noise ratio (SNR)^58,59^. Based on the intrinsic mathematical property of the correlation function, this algorithm can be used to realize two functions. One is the symmetric correlation peak generated by the auto- or cross-correlation between the BGSs, which enables a much easier precise calculation for the main peak location. The other function is the position or frequency shift of the correlation peaks for different strains, corresponding to changes in BFS or strain.

**Fig. 5:**
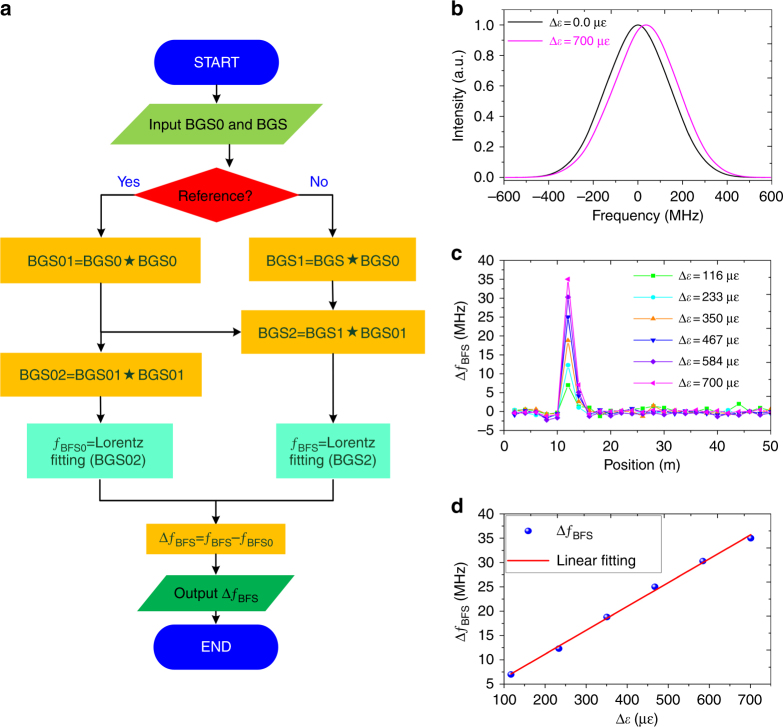
The strain change demodulation process. **a** The demodulation flow chart based on a twice-correlation algorithm; ⋆ represents the cross-correlation operator. **b** The reference BGS and the random BGS are processed by this algorithm, resulting in two symmetrical profiles. **c** The demodulated BFS changes along the 50-m PMF. **d** Change in BFS versus change in strain

The demodulation flow chart for this algorithm is shown in Fig. 5a. First, a measured BGS in the loose region is selected as the reference BGS0, and then, its first autocorrelation peak BGS01 and second autocorrelation peak BGS02 (black line in Fig. 5b) are computed. The twice-correlation results for the measured BGSs are given by

5 $${\rm{BGS2}} = \left( {{\rm{BGS}} \star {\rm{BGS0}}} \right) \star {\rm{BGS01}}$$

where ⋆ represents the cross-correlation operator. It can be seen that both correlation peaks BGS02 and BGS2 (e.g., magenta line for ∆*ε* = 700 με) show more symmetrical profiles and higher SNR compared to the original BGSs in Fig. 4b. Next, the correlation peaks BGS02 and BGS2 are Lorentzian or Gaussian fitted to calculate their central frequency *f*_BFS0_ and *f*_BFS_. Finally, the output of this flow chart is the BFS change

6 $$\Delta f_{{\rm{BFS}}} = f_{{\rm{BFS}}} - f_{{\rm{BFS0}}}$$

As a result, the distributed BFS changes for different strain measurements are shown in Fig. 5c; the data show that the demodulated BFS change in the strain segment at 10–12 m is clearly migrated away from a value of approximately zero in the non-strain segment. The strain dependences for the demodulated BFS changes are plotted in Fig. 5d, which reveals a good linearity with a fitting strain coefficient of 0.049 MHz/με.

### Dynamic strain measurements

To test the dynamic measurement performance, three different types of dynamic strain are established as follows:

#### Periodic mechanical vibration measurement

A 2-m segment located at a position of 44–46 m for a 50-m FUT is driven by an electro-motor to induce a periodic mechanical vibration for the test. The frequency-changing rate *η* is set to 20 MHz/ns, while the duration for the short optical chirp segment is set to 20 ns. For data acquisition, the original measured time trace for the Brillouin signal with no averaging can be collected via the fast-frame mode while setting the sampling rate to 25 kHz (corresponding to a period of 40 μs).

**Fig. 6:**
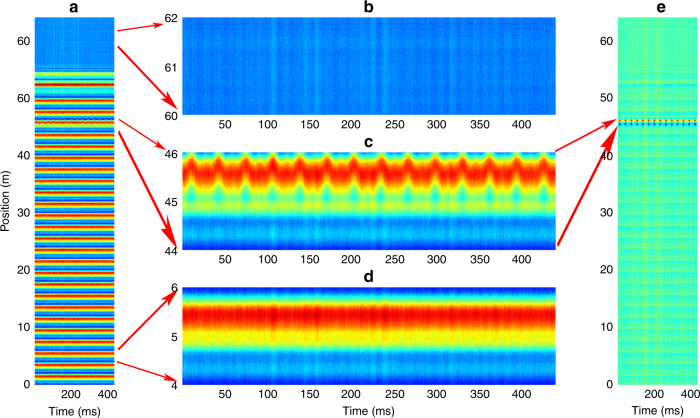
The position-time response for the periodic mechanical vibration. **a** The time evolution for the distributed BGS (the value is increased from blue to red). **b** The background pattern at the segment of 60–62 m. **c** The time evolution for the BGS at the segment of 44–46 m. **d** The time evolution for the BGS at the segment of 4–6 m. **e** The time evolutions for the distributed BGSs are processed by subtracting the initial distributed BGSs at time *t* = 0

**Fig. 7:**
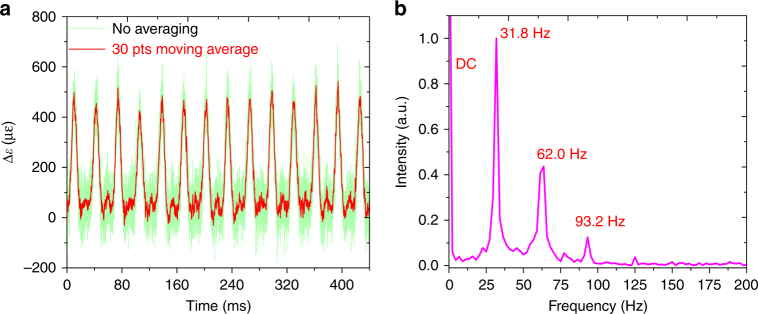
The time and frequency distribution of the periodic mechanical vibration. **a** The time evolution for the strain changes (green line) with no averaging, which represents the waveform for the periodic mechanical vibration, and 30-points moving average result (red line). **b** The frequency spectrum of the vibration

According to the order of frame and time, the time trace is reshaped with frame length *N*_seg_ = 3200(>2*nL*/*c*⋅5GS/s), forming a 3200 × 11000 array. The time evolution for the distributed BGS is shown in Fig. 6a. The segment of 60–62 m without signal, as shown in Fig. 6b, corresponds to a region where no sensing fiber is present. As shown in Fig. 6c, the BGSs at the vibrated segment of 44–46 m are position-shifted over time, while the BGSs at the loose segment of 4–6 m remain unchanged in Fig. 6d. Subsequently, by subtracting the initial distributed BGSs at time *t* = 0, the vibration position span and periodic patterns are rapidly processed, which can then be clearly located as demonstrated in Fig. 6e, which is suitable for real-time identification of vibration locations. The green line in Fig. 7a shows the time evolution for the strain change at the segment of 44–46 m demodulated by the “twice-correlation algorithm,” with the red line showing the result of a 30-points moving average, which corresponds to the waveform of the periodic mechanical vibration. The frequency spectrum for this waveform is obtained through fast Fourier transform, as shown in Fig. 7b, which contains a fundamental frequency component of 31.8 Hz with harmonics of 62.0 and 93.2 Hz.

#### Mechanical shock measurement

**Fig. 8:**
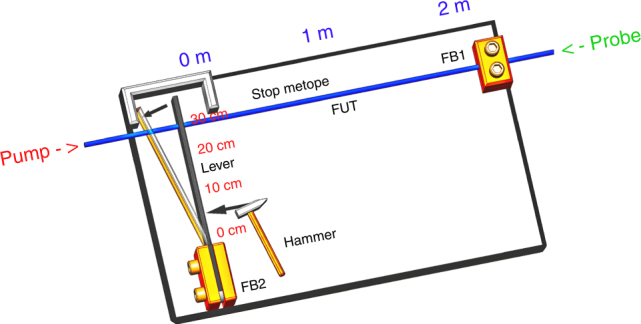
Schematic diagram for the mechanical shock measurement

A mechanical shock measurement is designed, as shown by the schematic diagram in Fig. 8. A 2-m segment of a 10-m FUT is selected to be stretched. The two end points for this section are fixed onto FB1 and the free end of a lever. In this experiment, through rapidly impacting the lever by a hammer, the free end of the lever will be rotated around FB2 so that the 2-m fiber section is quickly stretched. Meanwhile, this mechanical shock is captured by the proposed OCC–BOTDA system in which the sampling rate is set at 2.5 MHz (corresponding to a period of 400 ns). The frequency-changing rate *η* is 15 MHz/ns, while the duration for the short optical chirp segment is set to 20 ns.

**Fig. 9:**
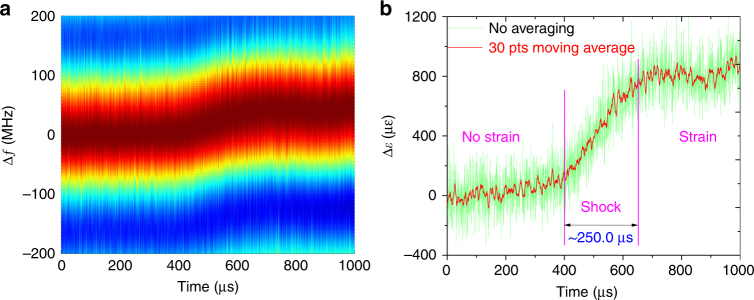
Time–frequency response for the mechanical shock measurement. **a** The BGS over time processed by the “twice-correlation algorithm” and **b** its strain change over time with no averaging (green line) and the result obtained for a 30-points moving average (red line)

The time evolutions for the BGSs in this section are first processed by the “twice-correlation algorithm,” with the result plotted in Fig. 9a. The demodulated strain change induced by mechanical shock is shown in Fig. 9b, where the green line shows the original result with no averaging and the red line shows the result obtained for a 30-points moving average. A shock time of ~250 μs with a strain change of ~800 με is demonstrated from the no-strain state at time *t* = ~400 μs to the strain state at time *t* = ~750 μs. This result shows tremendous potential for capturing fast processes, e.g., an explosion process. The standard deviation for the result is ~120 με with no averaging, while it decreases to 35 με for 30-points moving averaging since the power magnification of EDFA2 decreases as the repetition rate of the pump pulse increases to the order of a MHz.

#### Switch event demo

A switch event is carried out to demonstrate the ultrafast acquisition capability of the OCC–BOTDA system. The frequency-changing rate *η* and the duration for the short optical chirp segment are set to 15 MHz/ns and 20 ns, respectively, as done previously. Its notion can be summarized by noting that the microwave frequency agility spans every adjacent optical chirp segment are set at 2.400–2.695 GHz and 2.380–2.675 GHz, resulting in a frequency shift of 20 MHz (i.e., a strain change of ~408 με), which can be used to simulate a quick BFS change over time. The repetition rate for the pump pulse can reach up to 6.25 MHz (corresponding to a period of 160 ns).

**Fig. 10:**
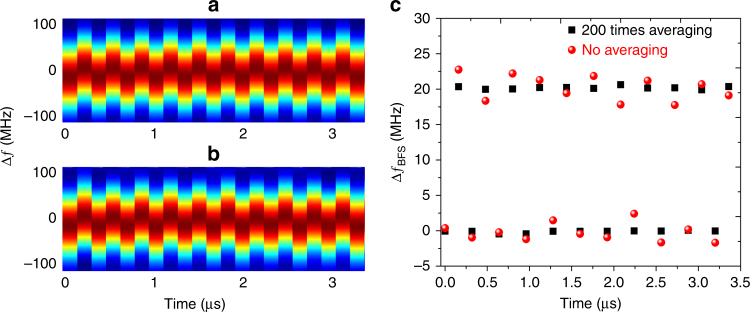
The time–frequency response for the switch event. The BGS over time is processed by the “twice-correlation algorithm” for **a** averaging 200 times and **b** no averaging. **c** The BFS changes over time for averaging 200 times (black scatter) and no averaging (red scatter)

The time evolution for the BGSs is processed by the “twice-correlation algorithm” at 200 times averaging and no averaging, as shown in Fig. 10a, b, respectively. Their BFS changes over time are shown in Fig. 10c, where the lower and the upper scatter sequences correspond to the frequency-agility spans of 2.400–2.695 GHz and 2.380–2.675 GHz, respectively. The frequency difference between the two scatter sequences is ~20 MHz, with the error for averaging 200 times and no averaging lying within ±0.5 MHz and ±2.5 MHz, respectively.

## Conclusions

In this work, an OCC–BOTDA scheme was theoretically proposed, numerically simulated, and experimentally verified for distributed ultrafast measurement with a maximum sampling rate of 6.25 MHz. The OCC probe wave is composed of several short optical chirp segments, which are generated through frequency modulation by use of the frequency-agile technique. When the frequency span between the OCC probe wave and the pump pulse overlaps the BFS for the sensing fiber, the distributed BGS along the fiber is revealed via the OCC probe wave in the time (or position) domain using only a single-shot pump pulse. The sampling rate for this proposed OCC–BOTDA is only restricted by the fiber length.

The performance for this proposed OCC–BOTDA is limited by the trade-offs among the frequency span, spatial resolution, and SNR. The effective scanning range is less than the frequency span due to the influence of a “ghost peak” and broadened BGS profile. As the frequency span increases for a wide-dynamic range, the SBS interaction region will be compressed, resulting in recovery of the BGS over a narrow region with a deteriorating SNR. The relatively lower SNR in this work may be due to the following factor: the AWG used in this work is composed by syncing a single output channel AWG with a high-sampling rate to an arbitrary-function generator with a lower-sampling rate, which results in electrical signal jitter with a root-mean-square of ~100 ps between the pump pulse and the OCC probe wave. For future work, a high-performance dual-channel AWG can be used to generate the pump pulse and the OCC probe wave to increase the SNR.

Additionally, the D&A module can be followed by a field-programmable gate array (FPGA) to realize a real-time response. We believe that the proposed OCC–BOTDA can provide dynamic or real-time distributed measurement for many potential applications, e.g., monitoring of large infrastructure and capturing an explosion process.
